# Assessing product adulteration of *Eurycoma longifolia* (Tongkat Ali) herbal medicinal product using DNA barcoding and HPLC analysis

**DOI:** 10.1080/13880209.2018.1479869

**Published:** 2018-07-28

**Authors:** Bashir Mohammed Abubakar, Faezah Mohd Salleh, Mohd Shahir Shamsir Omar, Alina Wagiran

**Affiliations:** a Department of Biotechnology & Medical Engineering, Faculty of Biosciences and Medical Engineering, UTM Skudai, Johor, Malaysia;; b Department of Biological Sciences, Bauchi State University Gadau, Bauchi, Nigeria;; c Department of Biosciences & Health Sciences, Faculty of Biosciences and Medical Engineering, UTM Skudai, Johor, Malaysia

**Keywords:** Chloroplastic rbcL, ITS2 barcode region, MEGABLAST

## Abstract

**Context:** *Eurycoma longifolia* Jack (Simaroubaceae) commonly known as Tongkat Ali is one of the most important plants in Malaysia. The plant extracts (particularly roots) are widely used for the treatment of cough and fever besides having antimalarial, antidiabetic, anticancer and aphrodisiac activities.

**Objectives:** This study assesses the extent of adulteration of *E. longifolia* herbal medicinal products (HMPs) using DNA barcoding validated by HPLC analysis.

**Materials and methods:** Chloroplastic *rbc*L and nuclear ITS2 barcode regions were used in the present study. The sequences generated from *E. longifolia* HMPs were compared to sequences in the GenBank using MEGABLAST to verify their taxonomic identity. These results were verified by neighbor-joining tree analysis in which branches of unknown specimen are compared to the reference sequences established from this study and other retrieved from the GenBank. The HMPs were also analysed using HPLC analysis for the presence of eurycomanone bioactive marker.

**Results:** Identification using DNA barcoding revealed that 37% of the tested HMPs were authentic while 27% were adulterated with the ITS2 barcode region proven to be the ideal marker. The validation of the authenticity using HPLC analysis showed a situation in which a species which was identified as authentic was found not to contain the expected chemical compound.

**Discussion and conclusions:** DNA barcoding should be used as the first screening step for testing of HMPs raw materials. However, integration of DNA barcoding with HPLC analysis will help to provide detailed knowledge about the safety and efficacy of the HMPs.

## Introduction

There has been a tremendous increase in the global use of herbal medicinal products (HMPs) due to their perceived health benefits (Abdullah et al. 2017). The general increase in the consumption of HMP may be due to the result of some factors such as their claimed health benefits, easy availability, perceived effectiveness and safety (Posadzki et al. 2013). It is estimated that about 5.6 billion people, approximately 80% of the world population relied on HMPs for their primary health care needs (Mohammad Azmin et al. 2016). The increase in demand has made the international trade for HMPs to become a lucrative business, thus presently, the global market is project to reach US$115 billion by the year 2020 (Ahmad et al. 2015).

Malaysia is not an exception to that as the demand of this HMP is also increasing due to the presence of diverse biodiversity of medicinal and flowering plants (Abubakar et al. 2017a). Malaysian National News Agency, BERNAMA report stated that the annual sales of traditional medicine from 2000 to 2005 increases tremendously from US$385 million (RM1 billion) to US$1.29 billion (RM4.5 billion) (Aziz and Tey 2009; Mohammad Azmin et al. 2016). This is mostly patronized among middle-age individuals so as to improve their health being and sexual libido (Hassali et al. 2012). It was also reported that about 69.4% of the Malaysian population use HMP in their whole lifetime (Mitha et al. 2013).

*Eurycoma longifolia* Jack (Simaroubaceae) commonly known as Tongkat Ali, is found in Southern Asian countries such as Malaysia, Indonesia, Vietnam, Myanmar, Cambodia and Thailand. *E. longifolia* extracts (particularly roots) have been reported to display a wide range of therapeutic properties such as antimicrobial, antimalarial, antidiabetic, antiulcer, cytotoxicity against cancerous cells (Bhat and Karim 2010; Nhan and Loc 2017) but the most famous is for aphrodisiac activities (Ismail et al. 2012). Several chemical studies have shown that *E. longifolia* contains important bioactive compounds such as canthin-6-one and β-carboline alkaloids, squalene derivatives, tirucallane-type triterpenes, biphenylneolignans and quassinoids such as eurycomanone, eurycolactone, eurycomalactone (Abubakar et al. 2017a). Nowadays, *E. longifolia* HMPs are more conveniently available in medicinal markets either in the form of raw crude root powder or as a capsule, tablet, or mixed with tea and coffee (Rehman et al. 2016).

The various conventional techniques such as organoleptic, microscopic and macroscopic analyses which have been used to identify medicinal plants have their own advantages and disadvantages (Smillie and Khan 2010). The major limitation of using these techniques is that they cannot effectively identify species in powder or a processed product form in which most of the HMPs are procured. However, the massive increase in demand of HMPs in addition to the absence of proper identification methods has made herbal industries to suffer from various adulterations such as substitution, contamination, the use of fillers (Techen et al. 2014). These type unethical activities are not only fraud but threat to the consumer’s health as the safety and efficacy of any HMPs is relied on the correct use of genuine plant material. Many DNA methods were developed for species identifications but due to their various limitations, they are unsuitable for rapid identification (Shi et al. 2017).

This prompted the introduction of a technique called DNA barcoding which utilizes short DNA sequence from the standard part genome for species identification. Contrary to the other types of techniques, DNA barcoding is accurate, cost effective, reliable, not influenced by age, environmental factors and it is not restricted by morphological characteristics of the medicinal plant material (Techen et al. 2014; Abubakar et al. 2017b).

However, despite the high level of demand and consumption of *E. longifolia* HMP, there is a complete absence or extreme scarcity in understanding the extents in which its HMPs have been adulterated. To the best of our knowledge, there has been no study that uses DNA barcoding to authenticate *E. longifolia* HMPs. Therefore, this study was designed to evaluate the authenticity of their HMPs using DNA barcoding and further validated by HPLC analysis so as to provide detailed information about their safety and efficacy as these are important towards quality control.

## Materials and methods

### Collection of plant and herbal product

The *E. longifolia* plant was obtained from Forest Research Institute Malaysia (FRIM), Selangor with a voucher ID number PID100317. The five-year-old root sample of *E. longifolia* was also purchased from FRIM. Eleven HMPs (nine capsules, one tea and one tablet) claimed to contain *E. longifolia* plants were purchased from different over-the-counter (OTC) retail shops in Jusco, Taman U, Plaza Angsana, and Larkin area of Johor, Malaysia. The HMP company name was not provided so as to promote anonymity, thus they are labelled as TAP-1 to TAP-11.

HPLC grade methanol was purchased from Fischer Scientific UK (Loughborough, UK), water for the HPLC analysis was treated with ultra water purification. Eurycomanone standard was purchased from Merck (Darmstadt, Germany). The purity of the standard was above 98%. All of the chemicals and reagents used in this study are of analytical grade.

### DNA extraction, PCR and sequence analysis

*E. longifolia* root material (approximately 100 mg) or 30 mg in the form of capsule or tea was first extracted using NucleoSpin^R^ plant II (Macherey-Nagel, Düren, Germany) according to manufacturer’s protocol. The yield and purity of the genomic DNA extracted were analysed using spectrophotometric nanodrop with an absorbance ratio (*A*_260/280_). The quality of DNA extracted from the fresh plant was checked on 1% (w/v) agarose gel electrophoresis. Two *rbc*L primers designated as full DNA barcode (FDB) and mini DNA barcode (MDB) were constructed according to Hamdan et al. (2013) and Fazekas et al. (2012), respectively. The FDB forward primers of *rbc*L were CTTGGC AGCATTCCGAGTA and TCACAAGCAGCAGCCAGTTC for reverse primers. The MDBs of *rbc*L were ATGTCACCACAA-ACAGAGACTAAAGC for forward primers and GTAAAATCA-AGTCCACCRCG for reverse primers. The primers used for ITS2 forward and reverse were GGGGCGGATATTGGCCTCCCGTGC and GACGCTTCT CCAGACTACAAT, respectively, as described by Chen et al. (2010).

PCR thermocycler programs for ITS2 were as follows: 95 °C, 2 min; 45 °C, 1 min; 72 °C, 2 min for 30 cycles. The PCR products were analysed on 1% (w/v) agarose gel stained with ethidium bromide. The PCR programme for *rbc*L (FDB) and MDB were also the same except the annealing temperature at 45 °C and 55 °C, respectively. The amplified PCR product was run on 1% agarose gel electrophoresis stained with ethidium bromide. PCR samples were prepared by adding genomic DNA template (20–100 ng/μL) to the reaction mixture containing 5 μL of 5× PCR buffer (Promega, Madison, WI), 2 μL of 25 mM MgCl_2_, 1 μL of 10 mM dNTPs mix, 1 μL of each forward and reverse primers (10 mM), 0.625 U of Taq DNA polymerase and sterile distilled water in a total reaction volume of 25 μL. The negative control was performed by adding sterile water to the PCR mixture. The amplified products of ITS2 and *rbc*L (FDB) were then purified using Wizard^®^ SV Gel and PCR Clean-Up System (Promega, Madison, WI) and ligated into pEasy-T3 cloning vector (Transgene Biotek, Hyderabad, Telangana) and transformed into an *E. coli* strain DH5α competent cells. The recombinant plasmids were identified using LB plate supplemented with IPTG, X-gal and ampicillin (Bio Basic, Markham, Canada). The positive recombinant DNA plasmids from the cloned were extracted using DNA QIAprep Spin Miniprep kit (Qiagen, Hilden, Germany). The recombinant plasmids were digested with *ECo*R1 and finally sent for sequencing at First Base Sdn Bhd (Seri Kembangan, Malaysia).

### DNA sequencing analysis

The reference DNA barcode material was developed using the generated sequences from the recombinant clones’ plasmid of *rbc*L (FDB) and ITS2. The generated sequences were trimmed and edited using BioEdit Software (v.7.2.5) and subsequently subjected to BLAST algorithm (Altschul et al. 1997) in the GenBank to identify the closest matching sequences. The sequences generated for the reference for *rbc*L (FDB) and ITS2 of these plants were used for the identification of adulterant in the *E. longifolia* HMP. The generated sequences were later deposited to National Centre for Biotechnology information (NCBI) database. For the authentication of *E. longifolia* HMPs using DNA barcoding, the forward and reverse sequences were manually trimmed using bioEdit software and used as a query sequences to compare to the GenBank nucleotides database using NCBI’s MEGABLAST program (http://blast.ncbi.nlm.nih.gov/Blast.cgi) for identification of the closest match (Morgulis et al. 2008).

The query sequences were taxonomically determined using minimum cutoff of 97% (Wallace et al. 2012). This is due to the higher similarity value between the query and data base sequences (to 100%), the closer the *e*-values to zero and the better the identification. HMPs are considered to pass a BLASTn criterion if the BLAST search resulted in *E. longifolia* with top match of 97% and above. Obtained sequences from the HMPs were also incorporated with the *E. longifolia* reference sequence established from this study and other sequences retrieved from the GenBank. A HMP is considered authentic and passed the neighbor-joining method based on the Kimura-2-Parameter Model (K2P) which was recommended as the standard barcoding method (Hollingsworth et al. 2009) if it formed a strongly supported monophyletic clade. The phylogenetic tree analyses were carried out using the program, MEGA 6 with 1000 bootstrap (BS) replicates (Tamura et al. 2013). HMPs were only considered truly authentic if they both passed BLASTn and neighbor-joining tree criteria.

### Chemical analysis using HPLC

HPLC chromatographic analysis of the HMPs was performed to determine the presence and concentration of bioactive marker in the *E. longifolia* root extract and tested HMPs which were identified as either authentic or substitution so as to confirm if there is correlation molecular genetic analysis.

### Preparation of stock solution for HPLC analysis

The stock concentration of eurycomanone standard solution was prepared at the concentration of 1 mg/mL in methanol. The eurycomanone reference standard was filtered through a 0.45 μm filter and stored at 4 °C prior to the HPLC analysis. The stock solution of eurycomanone was further diluted to the concentration range 0.5–0.01325 mg/mL.

### Preparation of *E. longifolia* roots extract and HMPs

The dried roots of *E. longifolia* was cut into 0.5 μm size and extracted with 150 mL methanol under sonication for 4 h as reported by Park et al. (2014). The solutes were filtered using Whatman No. 1 filter paper. The extract was also concentrated using rotary evaporator and dried using freeze drier to form powder. The *E. longifolia* root extracts and HMPs were prepared at the concentration of 1 mg/mL in HPLC grade methanol at room temperature with sonication for 15 min.

### HPLC method validation

To monitor the performance of the developed HPLC methods, some parameters were validated in order to judge whether the developed method is consistent, adequate and reliable for its intended purpose. The HPLC methods were fully validated for their linearity, accuracy, precision, limit of detection (LOD) and limit of quantification (LOQ) (ICH 2005; Khari et al. 2014). For the standard curve, the ratio of the peak area was plotted on the *y* axis while the concentration of the analytes was plotted on the *x* axis. The linearity was evaluated by analysing the eurycomanone standard at different concentrations (0.5. 025, 0.125, 0.0625 and 0.01325 mg/mL). Accuracy was ascertained by using three different concentration levels: 0.5. 025 and 0.125 mg/mL (*n* = 3) and the percentage recovery was calculated. The precision of the method was performed by determining the repeatability (intra-day precision) and intermediate precision (inter-day precision). The repeatability precision was evaluated by analysing six replicates of the same concentration of eurycomanone (0.5 mg/mL) within a day while the intermediate precision was assessed by analysing the same individual sample on three consecutive days. The result was calculated as %RSD. The LOD (which is calculated as 3.3 times the standard deviation of intercept divided by the slope) and LOQ (10 times the standard deviation of intercept divided by the slope) were assessed on the basis of a standard linear regression method (Basar et al. 2014).

### Quantification of eurycomanone in *E. longifolia* root extracts and HMPs

High performance liquid chromatography (HPLC) analysis was performed using 1260 infinity HPLC system (Agilent Technology, Santa Clara, CA) equipped with binary pump, degasser, 96-well plates autosampler and DAD detector. The chromatographic separation was carried out using Zorbax Eclipse XDB-C18 column (4.6 × 150 mm, 5 μm). The mobile phase comprises of isocratic mixture of methanol and water in the ratio of 85:15 (v/v) with a flow rate of 1.0 mL/min and the column temperature was maintained at 30 °C. A sample of 5 μL was injected and the wavelength was set at 254 nm. The purity of the eurycomanone compounds was assured from chromatograms, and the plant root extracts and HMPs were analysed for the presence of the eurycomanone. The calibration curve of eurycomanone was used for the quantification of eurycomanone content in the *E. longifolia* root exacts and HMPs using the linear regression equation. The results were calculated as % w/w using the formula: (calculated concentration/Loaded concentration × 100% (Memon et al. 2015).

## Results and discussion

### DNA extraction and amplification of DNA barcode

**Figure 1.**
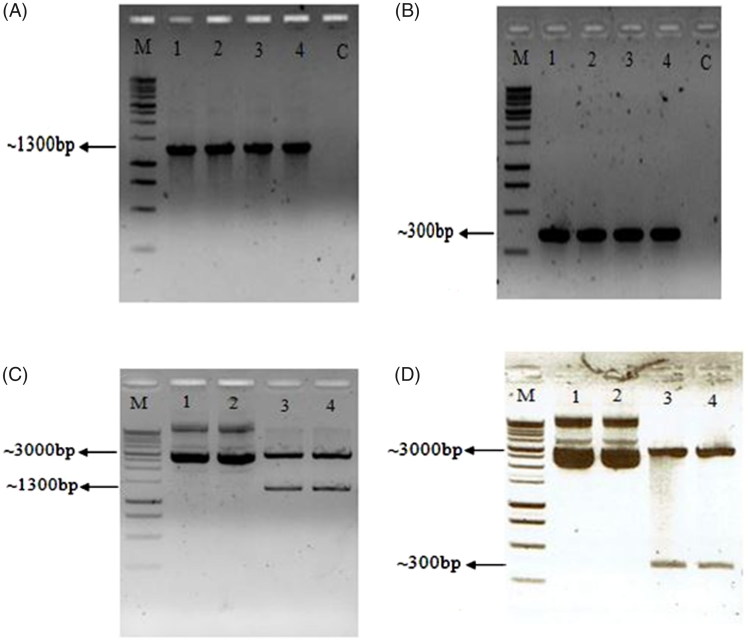
Agarose gel electrophoresis of several *rbc*L PCR (A) and ITS2 fragment (B). Lane C shows negative control. The successful recombinant plasmid of PEasy-*rbc*L (C) and PEasy-ITS2 recombinant plasmid (D) is shown. 1 kb DNA ladder (Promega, Madison, WI).

The extraction of genomic DNA from the fresh root part of *E. longifolia* showed *A*_260_/_280_ absorbance ratios above 1.6 which indicated high purity according to Cheng et al. (2014). The amplification of *rbc*L (FDB) and ITS2 fragments was successful production of distinct bands at an appropriate size of 1300 and 300 bp, respectively (Figure 1(A,B)). Analysis of restriction enzyme digestion of pEasy-T3:*rbc*L and pEasy-T3:ITS2 showed the presence of two distinct bands (Figure 1(C,D); lanes 3 and 4) of backbone vector (pEasy-T3) and the insert, *rbc*L (FDB) or ITS2 indicating that the two regions were successfully cloned. The sequence result of the pEasy-T3 cloned plasmid of *E. longifolia* revealed that the *rbc*L and ITS2 fragments have a sequence length of 1346 and 313 bp, respectively. The sequences generated for *rbc*L (FDB) and ITS2 have 99% similarities with those in the GenBank. The sequences generated were deposited in the GenBank with the accession number of MF509195 and KY264053 for *rbc*L (FDB) and ITS2, respectively.

### Authentication of *E. longifolia* HMPs

The spectrophotometer result of the yield and purity of the genomic DNA extracted from the *E. longifolia* HMPs using the Nucleospin Plant II protocol indicated variability reading with respect to the quality and quantity of product. In terms of yield, the highest concentration was obtained in TAP-2 herbal product (119.1 ng/µL) while the lowest was recorded in TAP-10 (5.3 ng/µL). On the other hand, the *A*_260/280_ reading showed that TAP-1, TAP-3, TAP-4, TAP-5, TAP-6, TAP-7, TAP-8 and TAP-9 fall within 1.6–1.7 (except for TAP-2, TAP-10 and TAP-11 which were below 1.6) where DNA extracted was of high quality.

Compared to fresh plant DNA, full length *rbc*L (FDA) primers have successfully amplified because the genomic DNA was mostly intact thus able to amplify longer sizes of product. However, this was not the case for HMPs as preliminary attempt to amplify the *rbc*L (FDB) failed. This was ascribed as a result of DNA degradation which occurred during the manufacturing processes. The presence of high amount of secondary metabolites such as polysaccharides, glycoproteins, polyphenolic compounds and pigments are derived from the raw materials within various tissues and organs of the plants and these have been proven to interfere with DNA extraction, amplification and sequencing (Zahra et al. 2016). In addition, the degradation of DNA at the primer annealing sites may also result in the unsuccessfully amplification of the selected DNA barcode regions, thus causing failed reaction (Newmaster et al. 2013). Large size region is always difficult to be amplified in fragmented or degraded DNA and this is due to the fact that the barcode region is larger than the average fragment size in DNA extract of the HMP to be amplified (Little and Jeanson 2013).

To overcome this problem, a new *rbc*L barcode with shorter length (MDB) was introduced into this study such as reported by Little and Jeanson (2013). Following PCR process, *rbc*L fragment was successfully amplified with a size approximately 500 bp from *E. longifolia* HMPs. Several reports have shown that shorter length DNA barcodes are used for the effective identification and authentication of HMPs as they are easy to be amplified (Little and Jeanson 2013; Little 2014).

Out of the total 11 samples of *E. longifolia* HMP extracted, the result showed amplifiable DNA was obtained from 7/11 (64%) of the tested HMPs. However, no amplification was detected from 4/11 (36%) of the tested HMPs after repeated attempts. This could be due to the result of the presence of inhibitors such as secondary metabolites including polyphenolic compounds, glycoprotein, polysaccharides which can interfere with the DNA extraction, amplification and sequencing (Zahra et al. 2016). These tested HMPs samples were designated as ‘No sequence’ and were not considered for further analysis. The results showed that ITS2 primers produced a highest amplification success 6/11 (55%) compared to *rbc*L (MDB) region 3/11 (27%).

### DNA sequence analysis and species identification in *E. longifolia* HMPs

**Figure 2.**
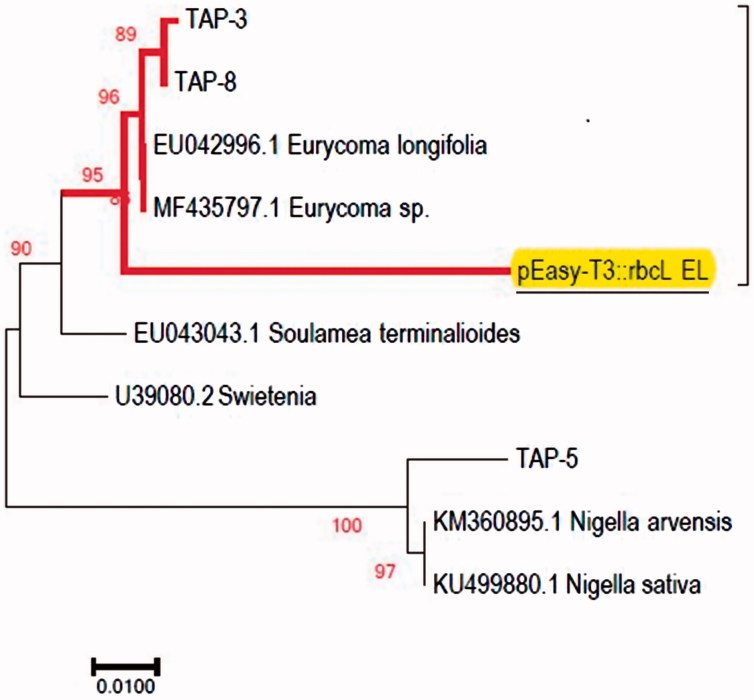
NJ tree of *rbc*L from *E. longifolia* HMPs compared to pEasy-T3::*rbc*L EL reference sequence of *E. longifolia* established from this work (underlined) and other sequences retrieved from the GenBank. Highlighted in bold is the strongly supported monophyletic clade. TAP: Tongkat Ali product. Numbers on the node for each branch are bootstrap scores for 1000 replicates (≥95).

When using *rbc*L (MBD) primers, the BLASTn result showed that only two of the tested HMP samples (TAP-3 and TAP-8) have high similarity to *E. longifolia* species above 97% (EU042996.1). The result of one of the tested HMP sample (TAP-5) has 99% similarities but with *Nigella sativa* (KU499880.1) or *Nigella arvensis* (KM360895.1) both belonging to the family Ranunculaceae (Table 1). Newmaster et al. (2013) reported that any substituted product may generate barcodes other than what was labelled on the tested HMP. Based on Figure 2, NJ analysis showed that only two of the tested HMPs samples (TAP-3 and TAP-8) formed a strongly supported monophyletic clade (BS = 95) with the *E. longifolia* reference sequence established from the present study (pEasy-T3::*rbc*L_EL) and other *E. longifolia* sequences retrieved from the GenBank database (EU042996.1 and MF435797.1) while TAP-5 clustered into another clade of *N. arvensis* and *N. sativa*. This result indicates that these two tested HMPs samples were truly authentic as they passed the two said criteria. The results indicated that *rbc*L can be reasonably amplified across a diverse set of plants but were not variable enough to discriminate species as also indicated by Michel et al. (2016).

**Table 1.** Sequence analysis of *rbc*L and ITS2 for the tested *E. longifolia* HMPs samples.

Sample code	Packaging label	HMP form	Barcode region	Identified species	Top similarities sequence from NCBI	Accession number	Barcode ID
TAP-1	*E. longifolia*	Capsule	ITS 2	*E. longifolia*	99%	KY553292.1	*Eurycoma longifolia*
			*rbc*L	NS	NS		
TAP-2	*E. longifolia*	Tablet	ITS 2	NS	NS		NS
			*rbc*L	NS	NS		
TAP-3	*E. longifolia*	Capsule	ITS 2	*E. longifolia*	99%	KY553292.1	*Eurycoma longifolia*
			*rbc*L	*E. longifolia*	99%	EU042996.1	
TAP-4	*E. longifolia*	Capsule	ITS 2	*Holcoglossum* sp.	97%	HQ452901.1	*Holcoglossum* sp.
			*rbc*L	NS	NS		
TAP-5	*E. longifolia*	Capsule	ITS 2	NS	NS		*Nigella arvensis* *Nigella sativa*
			*rbc*L	*Nigella arvensis* *Nigella sativa*	99%	KM360895.1 KU499880.1	
TAP-6	*E. longifolia*	Capsule	ITS 2	*E. longifolia*	97%	KY553292.1	*Eurycoma longifolia*
			*rbc*L	NS	NS		
TAP-7	*E. longifolia*	Tea	ITS 2	*Ficus deltoidea*	98%	HM159448.1	*Ficus deltoidea*
			*rbc*L	NS	–		
TAP-8	*E. longifolia*	Capsule	ITS 2	*E. longifolia*	99%	KY553292.1	*Eurycoma longifolia*
			*rbc*L	*E. longifolia*	99%	EU042996.1	
TAP-9	*E. longifolia*	Capsule	ITS 2	NS	NS		NS
			*rbc*L	NS	NS		
TAP-10	*E. longifolia*	Capsule	ITS 2	NS	NS		NS
			*rbc*L	NS	NS		
TAP-11	*E. longifolia*	Capsule	ITS 2	NS	NS		NS
			*rbc*L	NS	NS		

**Figure 3.**
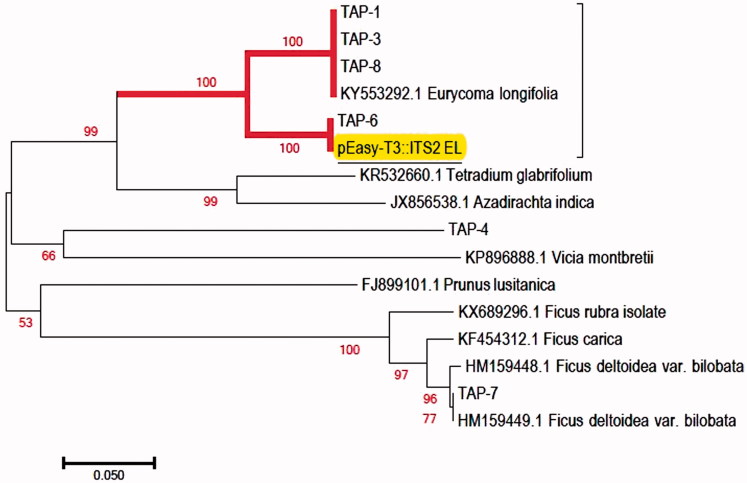
NJ tree of ITS2 from *E. longifolia* HMPs compared to pEasy-T3::ITS2 EL reference sequence of *E. longifolia* species established from this work (underlined) and other sequences retrieved from the GenBank. Highlighted in bold is the strongly supported monophyletic clade. TAP: Tongkat Ali Product. Numbers on the node for each branch are bootstrap scores for 1000 replicates (≥100).

The BLASTn result of the ITS2 barcoding regions showed that four of the tested HMPs samples (TAP-1, TAP-3, TAP-6 and TAP-8) matched with the expected *E. longifolia* above 97% (Table 1). However, the BLASTn result of two of the tested HMPs (TAP-4 and TAP-7) revealed that they contained the DNA barcode from other species contrary to what was labelled sample. TAP-4 has 97% similarity with *Holcoglossum* sp. (HQ452901.1). TAP-7 has 98% similarity with *Ficus deltoidea* (Moraceae) (HM159449.1). Based on Figure 3, NJ analysis of the ITS2 region of *E. longifolia* showed that four tested HMPs samples (TAP-1, TAP-3, TAP-6 and TAP-8) formed a strongly supported monophyletic clade (BS = 100) with the *E. longifolia* reference sequence established from the present study (pEasy-T3::ITS2_EL) and other *E. longifolia* sequence retrieved from the GenBank database (KY553292.1). This result indicates that the four tested HMPs samples were truly authentic as they passed the two said criteria (BLASTn and NJ tree analysis). However, two of the tested HMP samples (TAP-4 andTAP-7) nested outside the reference sample clade indicating that the HMPs contained other plant species contrary from those declared on their packaging labels.

The NJ result demonstrated that TAP-5 clustered with *N. arvensis* and *N. sativa* plant species. The *N. arvensis* was a native of Central and South East Europe and Near East Region and Northern Africa and there was no report of its cultivation in Malaysia (Havlik et al. 2006), this makes it unlikely to be the species substituted unless the manufacturer brought it from other country. NJ result was consistent with that of BLASTn results which revealed that the tested HMPs that contain *E. longifolia* cluster together, whereas those that do not contain *E. longifolia* contrary to what was on the packaged label clustered into their own clade. Therefore, BLASTn and NJ tree can be successfully used to distinguish HMPs that contain *E. longifolia* and those that do not.

The findings of the DNA barcoding in the present study indicated that four of the tested HMPs (TAP-1, TAP-3, TAP-6 and TAP-8) are authentic based on BLASTn and NJ tree criteria while three of the tested HMPs (TAP-4, TAP-5 And TAP-7) were possibly adulterated. According to Zhao et al. (2013), any new method for identifying traditional medicines should focus on accuracy, digitization, repeatability, simplicity and practicality. The DNA barcoding identification technique has important significance compared with the traditional methods. It can identify species in the form of dried herbs, leaves or roots because it is not restricted by morphological characteristics. Our findings showed that the ITS2 was the most promising DNA barcode for the authentication of *E. longifolia* HMP and this could be attributed as a result of their short size.

An ideal marker should be short enough to be amplified even in degraded DNA, produce high sequence quality and most have a powerful variability power to discriminate between samples (Vijayan and Tsou 2010; Li et al. 2015). Even though the *rbc*L barcode was successfully amplified in some of the *E. longifolia* HMPs, the ITS2 region was the most amplified region when compared. The ITS2 region has been confirmed by many researchers as one of the best DNA barcode region for the identification and authentication of medicinal plants (Gao et al. 2010; Han et al. 2016). In one of the most extensive study of DNA barcoding in medicinal plants reported by Chen et al. (2010), the ITS2 was shown to be the most suitable region for identification of medicinal plants. Their study showed that out of 6600 plant samples and their closely related species analysed, ITS2 region was able to identify 92.7% of the total sample. Other reports by He et al. (2012) also came with similar conclusion after analysing 109 samples using five DNA barcode regions, the ITS or ITS2 region was found to be the best among the other regions tested. The current study was the first to use a DNA barcode molecular identification technology to distinguish *E. longifolia* and its adulterants and this study expanded the application of the ITS2 sequence to the medicinal plant field especially in Malaysia. In conclusion, ITS2 as a DNA barcode would broaden our understanding of plant resource classification and phylogenetics.

The malpractice of labelling on the products showed a form of fraud because the consumer believed that they get the benefit of the said product but would not. For example, Olivar et al. (2016) documented that *Vitex negundo* (Lamiaceae) was replaced with different types of contaminant in which some of them are still medicinal plants (*Moringa olifeira* (Moringaceae) and *Mormodica charantia* (Cucurbitaceae)) after species identification using DNA barcoding. A similar occurrence was also found in the present study where TAP-7 and TAP-5 contained others species such as *F. deltoidea* and *N. sativa,* respectively, which have no aphrodisiac properties according to Bunawan et al. (2014) and Al-Adhroey et al. (2010). The *Holcoglossum* sp. which was the substituted species detected in TAP-4 HMPs was far more alarming because this flowering plant belonging to the family Orchidaceae (Xiang et al. 2011) does not have any therapeutic value. This may be an indirect or unintentional form of adulteration which involves the use of inferior or poor quality material in order to maximize profit (Satheeshkumar et al. 2016). Authentication methods maybe challenging in commercial product with incorrect species because the forms were changed, for example powdered in capsule or dried plant material in tea, which does not preserve morphological characteristics as well as lose their aromas due to volatile evaporation. However, the use of suitable extraction DNA method that produces high purity DNA should be able to amplify even from minute DNA samples. Thus, the NJ tree analysis in the present study clearly distinguished between *E. longifolia* and other species. Overall, this study demonstrated that ITS2 was efficient and effective than *rbc*L.

### Validation of the authenticity of *E. longifolia* HMPs using HPLC analysis

In order to confirm that the developed analytical method employed is reliable, adequate and consistence, the method of validation was carried out. In the present study, a standard solution of eurycomanone was used to determine the linearity, accuracy, precision, LOD and LOQ of the developed method. The calibration curve of eurycomanone was linear over the concentration range of 0.5–0.01325 mg/mL producing a linear regression equation of *y* = 72894*x* – 424.66 where *x* is the concentration of the eurycomanone (mg/mL) and *y* is the corresponding peak area. The correlation coefficient (*r* ^2^) was found to be 0.999 which indicates excellent correlation is obtained with good linearity. High recovery values fell within the range 100.14–102.37% at three different levels of concentration with acceptance %RSD of not more than 2% at each level (0.09–0.118%). The precision of the method was also demonstrated to be acceptable. The %RSD value of repeatability (intra-day) and intermediate precision (inter-day) were found to be 0.21 and 0.369%, respectively, both are less than 2% indicating satisfactory precision. The little differences that existed between %RSD values for intra-day and inter-day precision indicate the method is reproducible. The result of the developed method showed that the LOD and LOQ were 0.023 and 0.078 mg/mL, respectively, indicating high sensitivity.

**Figure 4.**
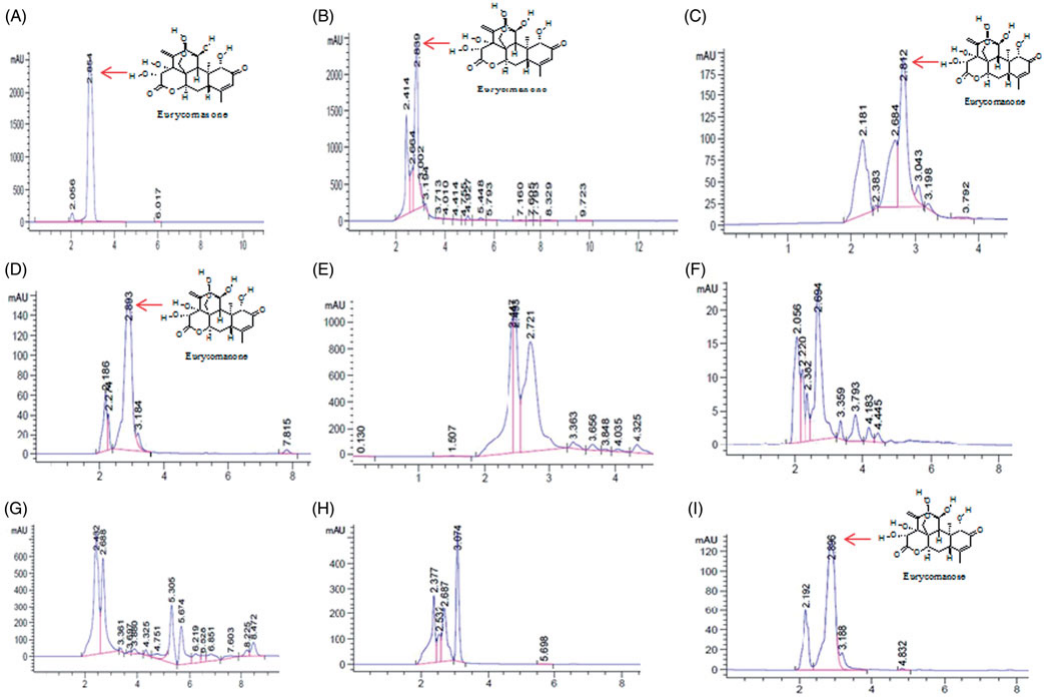
HPLC chromatogram of standard eurycomanone (A); HPLC chromatogram of *E. longifolia* root extract (B); HPLC chromatogram of *E. longifolia* HMPs (C–H and I for TAP-1, TAP-3, TAP-4, TAP-5, TAP-6, TAP-7 and TAP-8, respectively).

The validated method HPLC-DAD was subsequently used in the analysis of eurycomanone content in the *E. longifolia* root extract and HMPs. For the validation of the authenticity of the HMPs, only those HMPs which were identified as either authentic or substituted using DNA barcoding are the ones further analysed using this method. So also, the validation was based on the presence of eurycomanone marker which is one of the most important and abundant bioactive compound present in this plant (Nhan and Loc 2017). The eurycomanone standard showed a peak area with a retention time of 2.854 min similar to that of root extract 2.839 and three tested HMPs (TAP-1, TAP-3 and TAP-8). Other tested HMPs (TAP-4, TAP-5, TAP-6 and TAP-7) were found to have different reading of retention time and the yield of eurycomanone was undetected (ND) (Figure 4).

The result of the HPLC analysis showed that the root extracts of the *E. longifolia* have the highest percentage level of eurycomanone (6.11%) when compared to other HMPs. The high amount of this expected bioactive compound in the root extracts may be attributed due to the fact the root of the *E. longifolia* used in the present study was five years old. Sarwat et al. (2006) reported that as the tissue of high plants grows older particularly in herbal medicinal plants, the secondary metabolites which are responsible for the therapeutics properties increase. Even for commercial value, the roots of *E. longifolia* which usually contained most of the bioactive are normally harvested after 4 years of cultivation (Bhat and Karim 2010). This result is in agreement with those obtained by Khari et al. (2014) which showed that the percentage yield of eurycomanone from the roots of *E. longifolia* varies between 1.05%, 2.6% and 7.2% depending on the extraction method used. For the HMPs, the eurycomanone level was found to be 0.2%, 0.5% and 0.7% in TAP-1, TAP-8 and TAP-3, respectively. The other reading of retention time also showed absence of eurycomanone (Table 2).

**Table 2.** The concentration (mg/mL) and level of eurycomanone (%) in *E. longifolia* root extracts and HMPs.

Sample no.	Dosage form	Retention time (min)	Peak area (mAU)	Eurycomanone conc. (mg/mL)	Eurycomanone level (%) (w/w)
Root extract	Powder	2.839	22,718	0.30	6.11
TAP-1	Capsule	2.812	1461	0.01	0.28
TAP-3	Capsule	2.893	2663	0.03	0.74
TAP-4	Capsule	2.721	ND	ND	ND
TAP-5	Capsule	2.694	ND	ND	ND
TAP-6	Capsule	2.688	ND	ND	ND
TAP-7	Tea	3.074	ND	ND	ND
TAP-8	Capsule	2.896	2305	0.02	0.50

ND: not detected.

### DNA barcoding and HPLC chromatogram fingerprint correlation

Combining the authentication of *E. longifolia* HMPs using DNA barcoding and HPLC chemical analysis, certain results were found to be in agreement with each other. That was the case of three tested samples (TAP-1, TAP-3 and TAP-8) which were identified as authentic using DNA barcoding which were also found to contain eurycomanone. So also, three of the tested HMPs (TAP-4, TAP-5 and TAP-7) which were identified as substitution using DNA barcoding were found to be devoid of eurycomanone (Table 3). The use of DNA method and HPLC analysis has been reported by Mihalov et al. (2000) and successfully identified different types of ginseng in their commercial products.

**Table 3.** Summary of the authentication of *E. longifolia* HMP using the HPLC analysis.

No.	Sample name	DNA barcoding result			HPLC analysis result		
		ITS2	*rbc*L	Result summary	Eurycomanone	Eurycomanone (% level)	Result summary
1	TAP-1	Yes	No	Authentic	Present	0.28	Authentic
2	TAP-3	Yes	Yes	Authentic	Present	0.74	Authentic
3	TAP-4	Yes	No	Substitution	ND	ND	Substitution
4	TAP-5	No	Yes	Substitution	ND	ND	Substitution
5	TAP-6	Yes	No	Authentic	ND	ND	Substitution
6	TAP-7	Yes	No	Substitution	ND	ND	Substitution
7	TAP-8	Yes	Yes	Authentic	Present	0.50	Authentic

ND: not detected.

**Figure 5.**
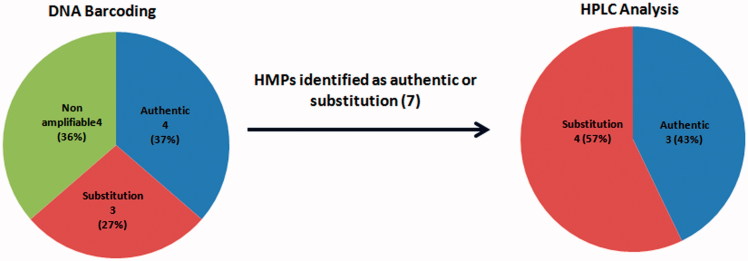
Correlation between DNA barcoding and HPLC analysis in the authentication of *E. longifolia* HMPs.

However, it is interesting to note that one of the tested samples (TAP-6) which was identified as authentic using the DNA barcode region was found not to contain the biochemical marker, eurycomanone. The possible reason for this is that eurycomanone which is one of the most important bioactive marker that is responsible for the pharmacological properties may be degraded as a result of external factors such as physiological or storage conditions or environmental factors such as altitude, temperature or other factors such as age of the plant, cultivation time, harvesting time, cultivation area, etc. This result was in accordance with the recent findings reported by Urumarudappa et al. (2016) that uses both molecular and chemical techniques to authenticate *Saraca asoca*, an important medicinal plant in India that is used for the treatment of various ailments such as leucorrhoea and other uterine disorders. In their study, they used DNA barcoding molecular technique together with an advanced form of chemical technique called NMR spectroscopy to authenticate the *Saraca asoca* in which they found that two samples (HAS107 and HAS424) are authentic using DNA barcoding but lack the expected chemical profile using the NMR chemical analysis. Figure 5 shows the correlation between the DNA barcoding and HPLC analysis results.

It is also notable to understand that out of the three tested samples which contain eurycomanone, none of them has met the minimum concentration of eurycomanone required by Department of Malaysian Standard (DMS). The amount of eurycomanone found in samples TAP-1, TAP-3 and TAP-8 was 0.28%, 0.74% and 0.50%, respectively, less than 0.8–1.5% as set by DMS (Khari et al. 2014). Previously, Norhidayah et al. (2015) reported that out of the 41 total number of *E. longifolia* HMPs tested, eurycomanone standard was only detected in 58.5% and only 26.8% fulfilled the set criteria. Hence low concentration of eurycomanone observed in the tested HMP in this study could indicate similar finding to their report.

The low concentration of eurycomanone content measured in these HMPs raised a possibility of another form of adulteration such as addition of undeclared synthetic phosphodiesterase-5 inhibitors (PDE-5i) drugs so as to enhance their efficacy. Several recent studies have shown that HMPs (advertised as natural products) which claimed to improve the quality of sexual life and libido were found to contain these types of synthetic drugs (Radu et al. 2015; Bujang et al. 2017; Fard and Akhgari 2018). Sildenafil (Viagra), tadalafil (cialis) and vardenafil are among the first synthetic PDE-5i drugs to be licensed by the US Food and Drug Administration (FDA) for the treatment of erectile dysfunction, the inability to maintain penile erection during intercourse (Rocha et al. 2016; Abubakar et al. 2017a). In Malaysia, these synthetic drugs fall in group B poisons found in the first schedule of the poison list. This group is under the control of Poison Act 1952 which stipulated that only authorized person such as registered medical practitioners and licensed pharmacists can supply and sell this type of drugs (Bujang et al. 2017). Therefore, possession of these types of drugs by unauthorized persons is illegal and punishable by the act. However, even though their usage is authorized but must be under medical supervision as their interaction with certain drugs can cause negative effects. Patients suffering from cardiovascular diseases such as hypertension, atherosclerosis are mostly treated with drugs containing nitrates, consequently combining such drugs with synthetic PDE-5i may drastically lower their blood pressure (Stief et al. 1998). Therefore, the unknowing consumption of such HMPs that contain undeclared synthetic PDE-5i may pose a serious health threat to the patient.

## Conclusions

The study showed that DNA barcoding is a quick and reliable method that can be successfully used for the authentication of *E. longifolia* HMPs. To identify the species specific of DNA barcoding, none restricted by morphological characteristics with decreasing cost of sequencing, this technique is becoming an important tool for the identification of herbal medicinal plants. The result of the study revealed that several *E. longifolia* HMPs sold in the Malaysian market are adulterated because the correct species was not identified. This study strongly suggests that there is an urgent need to utilize DNA barcoding to identify species adulterant, as this could serve as an avenue for the production of high quality HMPs. Therefore, DNA barcoding should be voluntarily used by herbal industries as the first screening step for the authentication of raw material used in the manufacturing of HMPs as most of them are exclusively sourced from the wild population. The use of DNA barcoding for the authentication of HMPs validated by chemical analyses will help to provide detailed knowledge about their safety and efficacy as these are very important towards quality control. Therefore, any unethical activity practice by herbal industries would be known and this will make them to produce products which are authentic, safe and of high quality, thereby restoring the consumers’ confidence and trust in HMPs.

## References

[CIT0001] Abdullah R, Diaz LN, Wesseling S, Rietjens IM 2017 Risk assessment of plant food supplements and other herbal products containing aristolochic acids using the margin of exposure (MOE) approach. Food Addit Contam. 34:135–144. 10.1080/19440049.2016.1266098 27892830

[CIT0002] Abubakar BM, Mohd FS, Wagiran A 2017a Chemical composition of *Eurycoma longifolia* (Tongkat Ali) and the quality control of its herbal medicinal products. J Appl Sci. 17:324–338.

[CIT0003] Abubakar BM, Mohd SF, Shamsir Omar MS, Wagiran A 2017b DNA barcoding and chromatography fingerprints for the authentication of botanicals in herbal medicinal products. Evid Based Complement Altern Med. 2017:1352948. 10.1155/2017/1352948 PMC5425840 28536641

[CIT0004] Ahmad F, Zaidi MAS, Sulaiman N, Majid FAA 2015 Issues and challenges in the development of the herbal industry in Malaysia. Prosiding Perkem. 10:227–238.

[CIT0005] Al-Adhroey AH, Nor ZM, Al-Mekhlafi HM, Mahmud R 2010 Ethnobotanical study on some Malaysian anti-malarial plants: a community based survey. J Ethnopharmacol. 132:362–364. 20723596 10.1016/j.jep.2010.08.006

[CIT0006] Altschul SF, Madden TL, Schäffer AA, Zhang J, Zhang Z, Miller W, Lipman DJ 1997 Gapped BLAST and PSI-BLAST: a new generation of protein database search programs. Nucleic Acids Res. 25:3389–3402. 9254694 10.1093/nar/25.17.3389 PMC146917

[CIT0007] Aziz Z, Tey N 2009 Herbal medicines: prevalence and predictors of use among Malaysian adults. Complement Ther Med. 17:44–50. 19114228 10.1016/j.ctim.2008.04.008

[CIT0008] Basar N, Talukdar AD, Nahar L, Stafford A, Kushiev H, Kan A, Sarker SD 2014 A Simple semi‐preparative reversed‐phase HPLC/PDA method for separation and quantification of glycyrrhizin in nine samples of *Glycyrrhiza glabra* root collected from different geographical origins. Phytochem Anal. 25:399–404. 24585378 10.1002/pca.2507

[CIT0009] Bhat R, Karim A 2010 Tongkat Ali (*Eurycoma longifolia* Jack): a review on its ethnobotany and pharmacological importance. Fitoterapia. 81:669–679. 20434529 10.1016/j.fitote.2010.04.006

[CIT0010] Bujang NB, Chee CF, Heh CH, Rahman NA, Buckle MJ 2017 Phosphodiesterase-5 inhibitors and their analogues as adulterants of herbal and food products: analysis of the Malaysian market, 2014–16. Food Addit Contamin A Chem Anal Control Expos Risk Assess. 34:1101. 10.1080/19440049.2017.1336674 28580889

[CIT0011] Bunawan H, Amin NM, Bunawan SN, Baharum SN, Mohd Noor N 2014 *Ficus deltoidea* Jack: a review on its phytochemical and pharmacological importance. Evid-Based Complement Altern Med. 2014:902734. 10.1155/2014/902734 PMC3977116 24772185

[CIT0012] Chen S, Yao H, Han J, Liu C, Song J, Shi L, Zhu Y, Ma X, Gao T, Pang X, et al. 2010 Validation of the ITS2 region as a novel DNA barcode for identifying medicinal plant species. PLoS One. 5:e8613. 20062805 10.1371/journal.pone.0008613 PMC2799520

[CIT0013] Cheng X, Chen X, Su X, Zhao H, Han M, Bo C, Xu J, Bai H, Ning K 2014 DNA extraction protocol for biological ingredient analysis of LiuWei DiHuang Wan. Genom Proteom Bioinformat. 12:137–143. 10.1016/j.gpb.2014.03.002 PMC4411345 24838067

[CIT0014] Fard HH, Akhgari M 2018 Analytical perspectives of chemical adulterants in herbal sexual enhancer drugs. J Pharm Pharmacogn Res. 6:45–53.

[CIT0015] Fazekas AJ, Kuzmina ML, Newmaster SG, Hollingsworth PM 2012 DNA barcoding methods for land plants. DNA barcodes. New York (NY): Springer; p. 223–252. 10.1007/978-1-61779-591-6_11 22684959

[CIT0016] Gao T, Yao H, Song J, Liu C, Zhu Y, Ma X, Pang X, Xu H, Chen S 2010 Identification of medicinal plants in the family Fabaceae using a potential DNA barcode ITS2. J Ethnopharmacol. 130:116–121. 20435122 10.1016/j.jep.2010.04.026

[CIT0017] Hamdan N, Samad AA, Hidayat T, Salleh FM 2013 Phylogenetic analysis of eight Malaysian pineapple cultivars using a chloroplastic marker (*rbc*L gene). J Teknol. 2:29–33.

[CIT0018] Han J, Pang X, Liao B, Yao H, Song J, Chen S 2016 An authenticity survey of herbal medicines from markets in China using DNA barcoding. Sci Rep. 6:18723. 26740340 10.1038/srep18723 PMC4703975

[CIT0019] Hassali MA, Saleem F, Shafie AA, Al-Qazaz HK, Farooqui M, Aljadhey H, Atif M, Masood I 2012 Assessment of general public perceptions toward traditional medicines used for aphrodisiac purpose in state of Penang, Malaysia. Complement Ther Clin Pract. 18:257–260. 23059442 10.1016/j.ctcp.2012.06.001

[CIT0020] Havlik J, Kokoska L, Vasickova S, Valterova I 2006 Chemical composition of essential oil from the seeds of *Nigella arvensis* L. and assessment of its actimicrobial activity. Flav Frag J. 21:713–717.

[CIT0021] He Y, Hou P, Fan G, Song Z, Arain S, Shu H, Tang C, Yue Q, Zhang Y 2012 Authentication of *Angelica anomala* Avé-Lall cultivars through DNA barcodes. Mitochondrial DNA. 23:100–105. 22397381 10.3109/19401736.2012.660924

[CIT0022] Hollingsworth PM, Forrest LL, Spouge JL, Hajibabaei M, Ratnasingham S, van der Bank M, Chase MW, Cowan RS, Erickson DL, Fazekas AJ, et al. 2009 A DNA barcode for land plants. Proc Natl Acad Sci USA. 106:12794–12797. 19666622 10.1073/pnas.0905845106 PMC2722355

[CIT0053] [ICH] International Conference of Harmonisation of Technical Requirements for registration of Pharmaceuticals for Human Use. 2005. ICH Harmonised Tripartite Guideline, Validation of Analytical Procedures: Text and Methodology Q2 (R1), Step 4 version. Genéva, Switzerland.

[CIT0054] Ismail SB, Mohammad W, Zahiruddin WM, George A, Nik HNH, Musthapa KZM, Liske E 2012 Randomized clinical trial on the use of Physta freeze-dried water extract of Eurycoma longifolia for the improvement of quality of life and sexual well-being in men. Evid Based Complement Alternat Med. 2012:429268. 23243445 10.1155/2012/429268 PMC3518798

[CIT0023] Khari N, Aisha AF, Ismail Z 2014 Reverse phase high performance liquid chromatography for the quantification of eurycomanone in *Eurycoma longifolia* Jack (Simaroubaceae) extracts and their commercial products. Trop J Pharm Res. 13:801–807.

[CIT0024] Li N, Shen Q, Wang J, Han C, Ji R, Li F, Jiang T 2015 Tetrodotoxin detection and species identification of pufferfish in retail roasted fish fillet by DNA barcoding in China. Food Addit Contam. 32:2148–2153. 10.1080/19440049.2015.1087056 26413972

[CIT0055] Little DP 2014 Authentication of Ginkgo biloba herbal dietary supplements using DNA barcoding. Genome. 57:513–516. 25495290 10.1139/gen-2014-0130

[CIT0025] Little DP, Jeanson ML 2013 DNA barcode authentication of saw palmetto herbal dietary supplements. Sci Rep. 3:3518. 24343362 10.1038/srep03518 PMC3865462

[CIT0026] Memon AH, Ismail Z, Al-Suede FSR, Aisha AF, Hamil MSR, Saeed MAA, Laghari M, Majid AMSA 2015 Isolation, characterization, crystal structure elucidation of two flavanones and simultaneous RP-HPLC determination of five major compounds from *Syzygium campanulatum* Korth. Molecules. 20:14212–14233. 26248073 10.3390/molecules200814212 PMC6331876

[CIT0056] Michel CI, Meyer RS, Taveras Y, Molina J 2016 The nuclear internal transcribed spacer (ITS2) as a practical plant DNA barcode for herbal medicines. J Appl Res Med Aromat Plants. 3:94–100.

[CIT0027] Mihalov JJ, Marderosian AD, Pierce JC 2000 DNA identification of commercial ginseng samples. J Agric Food Chem. 48:3744–3752. 10956181 10.1021/jf000011b

[CIT0028] Mitha S, Nagarajan V, Babar MG, Siddiqui MJA, Jamshed SQ 2013 Reasons of using complementary and alternative medicines (CAM) among elderly Malaysians of Kuala Lumpur and Selangor states: an exploratory study. J Young Pharm. 5:50–53. 24023454 10.1016/j.jyp.2013.05.002 PMC3758076

[CIT0029] Mohammad Azmin SNH, Abdul Manan Z, Wan Alwi SR, Chua LS, Mustaffa AA, Yunus NA 2016 Herbal processing and extraction technologies. Sep Purif Rev. 45:305–320.

[CIT0030] Morgulis A, Coulouris G, Raytselis Y, Madden TL, Agarwala R, Schäffer AA 2008 Database indexing for production MegaBLAST searches. Bioinformatics (Oxford, England). 24:1757–1764. 10.1093/bioinformatics/btn322 PMC2696921 18567917

[CIT0031] Newmaster SG, Grguric M, Shanmughanandhan D, Ramalingam S, Ragupathy S 2013 DNA barcoding detects contamination and substitution in North American herbal products. BMC Med. 11:222. 24120035 10.1186/1741-7015-11-222 PMC3851815

[CIT0032] Nhan NH, Loc NH 2017 Production of eurycomanone from cell suspension culture of *Eurycoma longifolia* . Pharm Biol. 55:2234–2239. 29130786 10.1080/13880209.2017.1400077 PMC6130563

[CIT0033] Norhidayah A, Vejayan J, Yusoff MM, Gambang K 2015 Detection and quantification of eurycomanone levels in Tongkat Ali herbal products. J Appl Sci. 15:999–1005.

[CIT0034] Olivar JEC, Alaba JPEP, Atienza JFM, Tan JJS, Umali MT, Alejandro GJD 2016 Establishment of a standard reference material (SRM) herbal DNA barcode library of *Vitex negundo* L.(Lagundi) for quality control measures. Food Addit Contam. 33:741–748. 10.1080/19440049.2016.1166525 26982211

[CIT0035] Park S, Nhiem NX, Van Kiem P, Van Minh C, Tai BH, Kim N, Yoo HH, Song J-H, Ko H-J, Kim SH 2014 Five new quassinoids and cytotoxic constituents from the roots of *Eurycoma longifolia* . Bioorg Med Chem Lett. 24:3835–3840. 25066952 10.1016/j.bmcl.2014.06.058

[CIT0036] Posadzki P, Watson L, Ernst E 2013 Contamination and adulteration of herbal medicinal products (HMPs): an overview of systematic reviews. Eur J Clin Pharmacol. 69:295–307. 22843016 10.1007/s00228-012-1353-z

[CIT0038] Radu GL, Popescu AM, Niculae CG, Raducanu AE, Onisei T 2015 Identification by liquid chromatography-mass spectrometry of herbal food supplements adulterated with PDE-5 inhibitors. Rev Chim. 66:1–5.

[CIT0039] Rehman SU, Choe K, Yoo HH 2016 Review on a traditional herbal medicine, *Eurycoma longifolia* Jack (Tongkat Ali): its traditional herbal medicine, evidence-based pharmacology and toxicology. Molecules. 21:331. 26978330 10.3390/molecules21030331 PMC6274257

[CIT0040] Rocha T, Amaral JS, Oliveira MBP 2016 Adulteration of dietary supplements by the illegal addition of synthetic drugs: a review. Comprehens Rev Food Sci Food Saf. 15:43–62. 10.1111/1541-4337.12173 33371574

[CIT0041] Sarwat M, Singh Negi M, Lakshmikumaran M, Kumar Tyagi A, Das S, Shankar Srivastava P 2006 A standardized protocol for genomic DNA isolation from *Terminalia arjuna* for genetic diversity analysis. Electron J Biotechnol. 9:86–91.

[CIT0042] Satheeshkumar N, Paul D, Lingesh A 2016 Liquid chromatography–mass spectrometry (LC–MS): approaches to adulterant detection in herbal products. Medicinal plants—recent advances in research and development. Singapore: Springer; p. 73–95.

[CIT0043] Shi Y, Zhao M, Yao H, Yang P, Xin T, Li B, Sun W, Chen S 2017 Rapidly discriminate commercial medicinal *Pulsatilla chinensis* (Bge.) Regel from its adulterants using ITS2 barcoding and specific PCR-RFLP assay. Sci Rep. 7:40000. 28059130 10.1038/srep40000 PMC5216359

[CIT0044] Smillie T, Khan I 2010 A comprehensive approach to identifying and authenticating botanical products. Clin Pharmacol Ther. 87:175–186. 20032974 10.1038/clpt.2009.287

[CIT0045] Stief CG, Uckert S, Becker AJ, Truss MC, Jonas U 1998 The effect of the specific phosphodiesterase (PDE) inhibitors on human and rabbit cavernous tissue *in vitro* and *in vivo* . J Urol. 159:1390–1393. 9507890

[CIT0046] Tamura K, Stecher G, Peterson D, Filipski A, Kumar S 2013 MEGA6: molecular evolutionary genetics analysis version 6.0. Mol Biol Evol. 30:2725–2729. 24132122 10.1093/molbev/mst197 PMC3840312

[CIT0047] Techen N, Parveen I, Pan Z, Khan IA 2014 DNA barcoding of medicinal plant material for identification. Curr Opin Biotechnol. 25:103–110. 24484887 10.1016/j.copbio.2013.09.010

[CIT0048] Urumarudappa SKJ, Gogna N, Newmaster SG, Venkatarangaiah K, Subramanyam R, Saroja SG, Gudasalamani R, Dorai K, Ramanan US 2016 DNA barcoding and NMR spectroscopy-based assessment of species adulteration in the raw herbal trade of *Saraca asoca* (Roxb.) Willd, an important medicinal plant. Int J Legal Med. 130:1457–1470. 27627901 10.1007/s00414-016-1436-y

[CIT0049] Vijayan K, Tsou C 2010 DNA barcoding in plants: taxonomy in a new perspective. Curr Sci. 99:1530–1541.

[CIT0050] Wallace LJ, Boilard SM, Eagle SH, Spall JL, Shokralla S, Hajibabaei M 2012 DNA barcodes for everyday life: routine authentication of Natural Health Products. Food Res Int. 49:446–452.

[CIT0051] Xiang X-G, Hu H, Wang W, Jin X-H 2011 DNA barcoding of the recently evolved genus *Holcoglossum* (Orchidaceae: Aeridinae): a test of DNA barcode candidates. Mol Ecol Resour. 11:1012–1021. 21722327 10.1111/j.1755-0998.2011.03044.x

[CIT0052] Zahra NB, Shinwari ZK, Qaiser M 2016 DNA barcoding: a tool for standardization of herbal medicinal products (HMPs) of Lamiaceae From Pakistan. Pak J Bot. 48:2167–2174.

[CIT0037] Zhao X, Pang S, Shan T, Liu F 2013 Applications of three DNA barcodes in assorting intertidal red macroalgal flora in Qingdao, China. J Ocean Univ China. 12:139–145.

